# Language Difficulty and Prior Learning Influence Foreign Vocabulary Acquisition

**DOI:** 10.3390/languages5010002

**Published:** 2019-12-27

**Authors:** Sayuri Hayakawa, James Bartolotti, Aimee van den Berg, Viorica Marian

**Affiliations:** Department of Communication Sciences and Disorders, Northwestern University, Evanston, IL 60208, USA

**Keywords:** foreign language acquisition, desirable difficulties, imageability, language similarity, vocabulary

## Abstract

When learning a foreign language, words that are the hardest to learn are often the easiest to forget. Yet, there is also evidence that more challenging learning contexts can lead to greater long-term retention. Here, we investigate the effect of language difficulty on vocabulary retention by teaching participants novel words that varied in both imageability and similarity to a known language over a period of four weeks. We found that easier words (high-imageability and familiar) were generally retained better than harder words (low-imageability and unfamiliar). However, when words were fully learned during training, the more difficult unfamiliar words were later recalled with higher accuracy than easier familiar words. The effect of language difficulty on vocabulary retention therefore varies depending on how well words were initially encoded. We conclude that greater challenges can reap greater long-term rewards so long as learners establish a strong foundation during initial acquisition.

## 1. Introduction

When learning a new skill, whether it be computer programming, tennis, or a foreign language, many of us have experienced a ‘honeymoon period,’ where the transformation from complete novice to competent newcomer prompts a rush of excitement and confidence. Just as common, however, is the ensuing loss of motivation after discovering how much further there is to go. What many learners fail to appreciate is that the times of struggle can be the most beneficial. Research on memory and learning has demonstrated that we often retain information better if it is encoded under conditions of *desirable difficulty* (Bjork 1994). For example, varying the learning context (Smith et al. 1978), distributing practice over time (Janiszewski et al. 2003), and introducing interference (Shea and Morgan 1979) have all been shown to improve retention of skills and knowledge over time. Unsurprisingly, more difficult learning conditions will often lead to reduced competence in the beginning (Soderstrom and Bjork 2015), suggesting that the tactics most effective for rapid initial gains may not be the most effective for long-term retention. However, given that learning is an incremental process, excess difficulty may hinder the development of a proper foundation. In the present study, we explore the impact of language difficulty on the initial acquisition and eventual retention of foreign language vocabulary. Though there is substantial evidence demonstrating that challenging learning procedures can have long-term benefits, the effects of difficulty originating from characteristics of the language itself are less consistent. Furthermore, the effects of content, as opposed to procedure-related features are less discussed in the task-difficulty literature, despite the fact that both the learning procedures and the material to be mastered could reasonably be expected to impact learners’ levels of engagement. We therefore examine the effects of word characteristics (i.e., imageability and similarity to known languages) on both acquisition and retention, with a particular focus on the ways in which the ultimate impact of language difficulty is moderated by levels of prior learning.

How easily individuals are able to initially learn foreign language vocabulary depends on a number of different factors. These include affective variables such as motivation (Gardner et al. 1985; MacIntyre 2002) and anxiety (Dewaele et al. 2008; MacIntyre and Gregersen 2012), the cognitive processes involved in utilizing different strategies (Laufer and Hulstijn 2001; Mokhtar et al. 2000; Oxford et al. 2004), and most pertinent to the present investigation, linguistic features of the words themselves (Crossley et al. 2016; de Groot and Hell 2005; Ellis and Beaton 1993; Peters 2019; Pichette et al. 2012; Puimège and Peters 2019). It has been demonstrated that words that share substantial phonological, semantic, and orthographic overlap with a known language are more easily learned than those that do not (Ringbom and Jarvis 2009). For instance, cognates, which share both semantic and phonological features (e.g., English ‘family’ and Spanish ‘familia’) will be more readily acquired than non-cognates (e.g., English ‘neighbor’ and Spanish ‘vecino/a’; (Lotto and de Groot 1998)). Even without such precise overlap, novel vocabulary is more easily learned when it is phonotactically similar to the native tongue (Bartolotti and Marian 2017; Storkel 2001), likely because learners can take greater advantage of existing linguistic knowledge. Another factor with robust effects on vocabulary acquisition is imageability. The ease with which a word evokes a mental image has been shown to facilitate vocabulary acquisition, both in L1 (Ma et al. 2009) and L2 (de Groot and Keijzer 2000; de Groot 2006; Ellis and Beaton 1993; van Hell and Mahn 1997), potentially because novel words can be anchored to not only the L1 translation, but also to their visual representations (i.e., ‘dual-coding theory’; (Paivio 1990)). Concreteness has similarly been shown to contribute to the ease of novel vocabulary acquisition using both explicit (Elgort and Warren 2014; Kaushanskaya et al. 2013; Mestres-Missé et al. 2009) and incidental (Puimège and Peters 2019) learning paradigms. In addition to potentially benefiting from greater imageability, concrete words may be easier to learn because they are more readily embedded in a context, which has been shown to facilitate comprehension (i.e., ‘context-availability theory’; (Schwanenflugel and Shoben 1983)). Imageability and concreteness are often highly correlated with each other (Altarriba et al. 1999), but it is worth noting that the two are dissociable, as highly concrete words can be difficult to imagine depending on individuals’ personal experiences with them (e.g., “armadillo”; see (Stadthagen-Gonzalez and Davis 2006)). Furthermore, though the terms concreteness and imageability are sometimes used interchangeably (Crossley et al. 2016; de Groot and Keijzer 2000; Peters 2019), there is evidence that they can have distinct effects for learning and recall (Bird et al. 2001; Richardson 1975).

Equally important to understanding the characteristics of language that facilitate or hinder initial acquisition are the factors that impact the eventual retention of previously learned words. Schmitt (2010) observes that before a word becomes ‘fixed’ in memory, vocabulary knowledge often fluctuates between states of learning and forgetting, and lexical knowledge (particularly word-form; see (van Zeeland and Schmitt 2013)) appears to be especially vulnerable to attrition relative to more rule-based linguistic knowledge such as grammar. As discussed, research on learning and memory in other domains has demonstrated that the strategies and factors that present a challenge during initial learning may in fact lead to greater retention in the long term (e.g., Potts and Shanks 2014; Shea and Morgan 1979; Soderstrom and Bjork 2015). Similarly, evidence suggests that novel words acquired through active and effortful learning processes may form more stable representations in memory relative to those that are acquired more passively ((de la Fuente 2002; Joe 1995; Paribakht and Wesche 1997); see (Laufer and Hulstijn 2001) involvement load hypothesis). In one study, Schneider et al. (2002) trained and tested native English speakers on English–French word pairs, either by having them produce backward translations from French to English (L2 to L1) or forward translations from English to French (L1 to L2). The authors found that while performance on a test immediately following initial training was worse for those completing the forward translation task, possibly due to the greater difficulty of producing an unfamiliar word, those completing the initially easier backward translation task had significantly greater attrition when tested a week later. Other manipulations of procedure-related task difficulty have yielded similar findings—for instance, Storm et al. (2014) found that native English speakers ultimately remembered more Swahili words if they had been repeatedly tested (with feedback) during training rather than passively studying the materials.

During early stages of learning, however, engaging in challenging training procedures (e.g., production and elaboration) can have a detrimental impact on ultimate attainment if learners are unprepared to meet the demands of the task (Barcroft 2002, 2004). The benefits of more effortful acquisition may additionally vary depending on the source of task difficulty, such as those stemming from learning procedures versus the language itself. Indeed, the long-term effects of difficulty resulting from word characteristics have received relatively less attention and have been somewhat inconsistent. Pichette et al. (2012) observed that, while initial performance was superior for concrete words, this advantage was no longer present a week later. Though there was no indication that abstract words were more likely to be recalled than concrete words (i.e., as might be expected for a *desirable difficulty* effect), their findings did indicate that the easier concrete words were subject to steeper rates of forgetting over the course of the week. On the other hand, Tagashira (2001) observed that there was a significantly greater drop in recall for abstract words compared to concrete words 4 days after learning. However, there was no further decrease in performance for either concrete or abstract words when tested a week later, suggesting that word characteristics may not affect retention once a stable state of encoding has been achieved. These results appear to be inconsistent with de Groot and Keijzer (2000) finding that the hardest words to learn are also the most likely to be forgotten (that is, the opposite of a *desirable difficulty* effect). Specifically, de Groot and Keijzer (2000) observed that both acquisition and retention of new words are greatly facilitated when novel information can be grounded in existing semantic knowledge (e.g., cognates and concrete words, as compared to non-cognates and abstract words^1^). Similarly, the ability to rely on familiar phonological information has been shown to benefit learning beyond the earliest stages by guarding against language attrition (de Groot 2006). Though the actual results obtained by Tagashira (2001) and de Groot and Keijzer (2000) differed in the long-term effects of concreteness on retention, their theoretical perspectives are, in fact, quite compatible. de Groot (2006) explains her findings using Atkinson (1972) conceptualization that novel vocabulary can be categorized as being in one of three states: ‘P,’ where knowledge of the word is relatively permanent and resistant to interference, ‘T,’ where the word is known on a temporary basis, but is unstable in memory, and ‘U,’ where the word is unknown. de Groot (2006) suggests that vocabulary that is more easily acquired, such as cognates and concrete words, is more likely to reach the relatively permanent ‘P’ state during initial encoding, making it less likely to be forgotten over time. Tagashira (2001) may be correct that attributes such as concreteness may have a minimal impact on retention once words have been stably encoded, but de Groot (2006) posits that it is precisely the likelihood of reaching such a state that is moderated by word characteristics. It is therefore possible that variable effects of task difficulty (both across studies investigating word characteristics, as well as for procedure- versus language-induced difficulty) may be at least partly explained by accounting for differences in the memory state that is reached during initial encoding. Specifically, it may be that more difficult words are less likely to reach the relatively permanent ‘P’ state compared to easy words, but that in cases where they are successfully encoded, the additional difficulty makes them all the more resistant to memory decay.

The present study attempts to bridge the literature examining procedure-induced *desirable difficulties* and the literature examining the impact of word characteristics on vocabulary retention. Specifically, we explore the effect of language difficulty while considering the degree to which each word was initially learned. Unlike prior studies, which have often operationalized learning as a percentage of all words that were fully remembered (e.g., de Groot and Keijzer 2000; de Groot 2006), the present study accounts for partial learning by assigning points for each letter that was accurately written in the correct position. Furthermore, we examine whether the trajectory of learning over time varies as a function of two different factors known to influence the difficulty of initial vocabulary acquisition: imageability and similarity to a known language. In this way, we examine (1) whether the impact of language difficulty on vocabulary retention is moderated by initial levels of learning (e.g., partial vs. complete initial recall), and (2) whether there are variable effects of difficulty resulting from semantic versus phonological characteristics of the study materials.

The effects of prior learning and language difficulty were assessed through the use of a paired-associates task. Despite growing consensus regarding the benefits of contextualized forms of vocabulary learning (see Godwin-Jones 2018), the use of a relatively simpler paired-associates task enabled us to minimize the influence of procedure-induced task difficulty that could interact with or overshadow the language-related variables of interest. Paired-associate learning is often assumed to promote rote-memorization tactics (Oxford and Crookall 1990) and is therefore used in comparison with procedures that require a deeper level of engagement (e.g., reading literature, (Herman 2003); semantic mapping, (Sagarra and Alba 2006)). Similar paired-associate translation tasks have been used to study word learning in general (e.g., Chow 2018; Kaushanskaya and Marian 2009), as well as the effects of word characteristics in particular (de Groot 2006; de Groot and Keijzer 2000; Kaushanskaya and Rechtzigel 2012). Although the relatively decontextualized nature of paired-associate learning may call the ecological validity of the task into question, repetition and memorization remain popular strategies among language learners (Ko and Goranson 2014), are still frequently used in language learning applications (Gunter et al. 2016; Wu 2015) and, in some cases, have been shown to be as or more effective than tasks with higher levels of involvement load (e.g., Khoii and Sharififar 2013; Sagarra and Alba 2006). For example, rote memorization and paired-associate learning may be especially effective for more advanced learners (van Hell and Mahn 1997), as well as for establishing connections between form and meaning (Kasahara 2011).

## 2. Materials and Methods

### 2.1. Participants

Participants included sixty-five English speakers (63 female), reporting an average English proficiency of 9.79 out of 10 (*SD* = 0.51), as assessed by the LEAP-Q (Marian et al. 2007), which corresponds to a C2 (mastery) level of proficiency using the Common European Framework (CEF; Council of Europe 2001). The mean age at the time of the experiment was 25.35 years (*SD* = 2.11), and the mean age of English acquisition was 0.53 years old (*SD* = 1.54). Approximately half (N = 36; 55.4%) of the participants reported experience with a language other than English. Among these participants, the average non-English proficiency was 5.23 out of 10 (*SD* = 2.41; roughly corresponding to a B1 (intermediate) level of the CEF), and the average age of non-English acquisition was 8.51 years old (*SD* = 6.19). Demographic and language background variables did not significantly differ between experimental conditions (all *p* > 0.05). All subjects gave their informed consent for inclusion before they participated in the study. The study was conducted in accordance with the Declaration of Helsinki, and the protocol was approved by the Ethics Committee of Northwestern University (IRB STU00023477).

### 2.2. Materials

In order to manipulate language difficulty, we created two artificial languages, each containing 48 words paired with English translations. Both artificial languages were paired with the same set of English words, half of which were classified as ‘high-imageability’ and half as ‘low-imageability’ based on standardized ratings obtained from the Bristol norms (Stadthagen-Gonzalez and Davis 2006). The Bristol norms are scaled from 100 to 700 with higher numbers indicating higher levels of imageability. Using the mid-point of 400 as the upper and lower limits for low- and high-imageability words, respectively, the final set of low-imageability native translations had a mean rating of 280 (*SD* = 48.75; Range = 154–346) and high-imageability stimuli had a mean rating of 629 (*SD* = 18.43; Range = 600–668). All novel words were composed of five letters with alternating vowels and consonants (CVCVC). Critically, one language was constructed to be phonotactically similar to English (‘Familiar’), while the other was relatively dissimilar (‘Unfamiliar’). Language difficulty was thus manipulated both between subjects (Familiar vs. Unfamiliar) as well as within (Low- vs. High-imageability). To create the languages, 10,000 novel words were randomly generated and their phonological forms were determined with the use of eSpeak speech synthesizer software (version 1.48.15 for Linux; (Duddington 2012)). Average bigram and biphone probabilities in English were calculated using CLEARPOND (Marian et al. 2012). These scores were then z-transformed and averaged to serve as an index of similarity to English. Novel words were classified as either ‘Familiar’ or ‘Unfamiliar’ based on their similarity ranking relative to real five-letter English words obtained from SUBTLEXUS (Brysbaert and New 2009). The ‘Familiar’ language only included novel words with English similarity scores at or above the 20th percentile and the ‘Unfamiliar’ language only included words below the 99th percentile.

### 2.3. Procedure

Participants were randomly assigned to learn either the familiar (N = 33) or unfamiliar language (N = 32). The two groups did not significantly differ from each other in gender, age, English proficiency, age of English acquisition, proportion of participants with other language experience, non-English proficiency (when applicable), or age of non-English acquisition (all *p* > 0.05). Learning was assessed over a four week period with two training sessions spaced one week apart and a surprise recall test two weeks after the second training session. All sessions were conducted in a large classroom setting under the supervision of the experimenter, with all participants tested at the same time. For the initial training session in week 1, participants were given 16 min to read and study a list of 48 foreign–English word pairs (e.g., naren–code) written on a sheet of paper. Immediately after studying, participants were once again presented with a sheet of paper listing only the English words and were given 6 min to write down their translations in the novel foreign language (i.e., forward translation). On week 2, participants completed a follow-up training, this time with 8 min to study the same list of word pairs and 6 min to complete the translation task. Lastly, on week 4, participants were given 8 min to complete the same translation task, but without studying the list beforehand. Each correct letter written in the correct position was assigned 0.2 points so that each word received an accuracy score ranging from 0 to 1.

## 3. Results

### 3.1. Word Imageability and Language Familiarity over Time

In order to more easily compare the present findings with those of similar prior studies (e.g., de Groot 2006; de Groot and Keijzer 2000; Schneider et al. 2002), we first examined the effects of language difficulty on word accuracy in each session without accounting for prior levels of learning on subsequent performance. Each word’s accuracy score was entered as the response variable in a linear mixed-effects regression with Familiarity (Familiar vs. Unfamiliar), Imageability (High vs. Low), and Session (1, 2, or 3) plus interactions as fixed effects and Subject and Item as random effects. The model was fitted utilizing the ‘lme4’ package (Bates et al. 2015) in the R environment (R Core Team 2015), and significance of fixed effects was tested using the Satterthwaite approximation for degrees of freedom.

**Figure 1.**
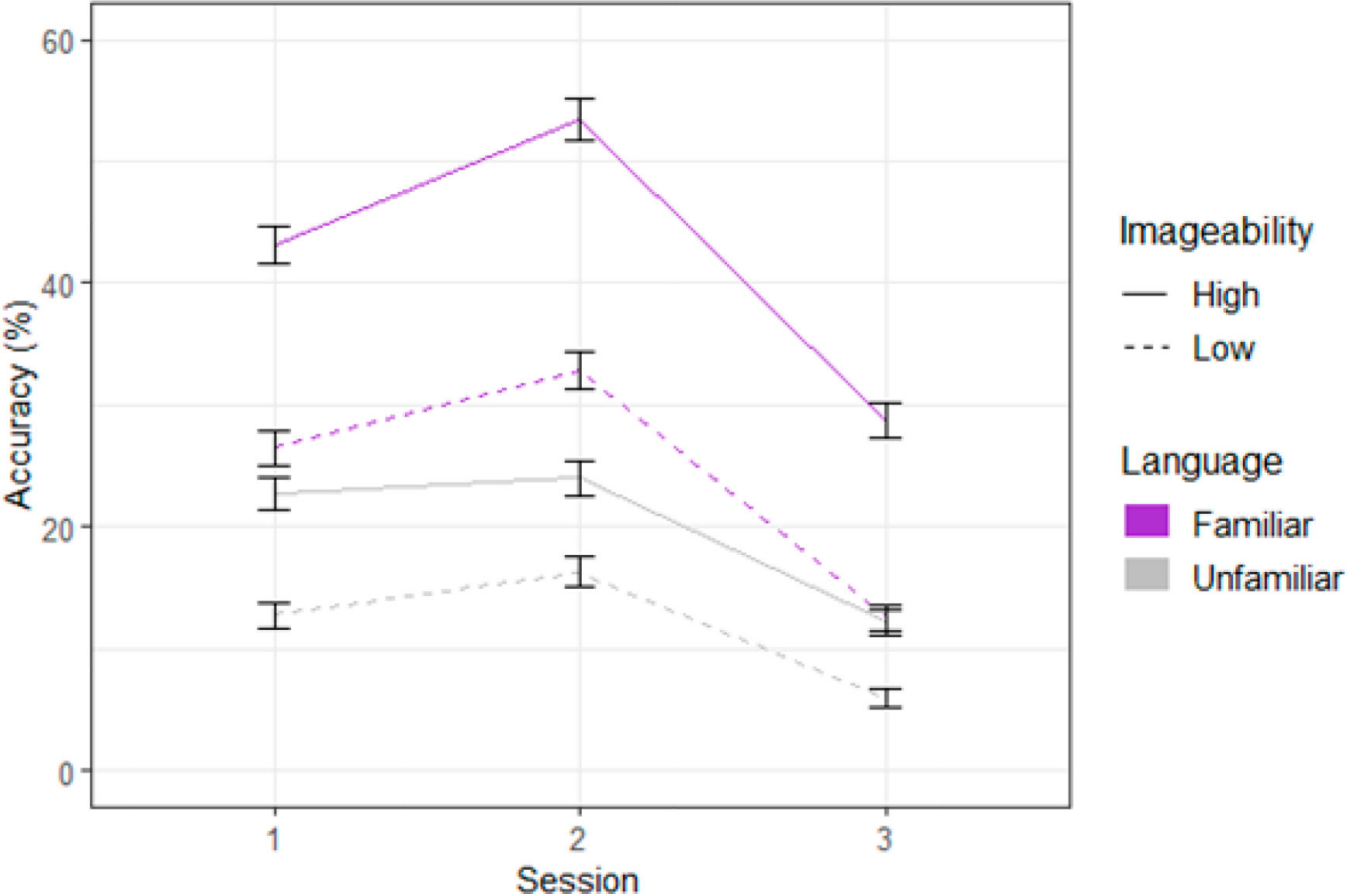
Effects of familiarity and imageability on accuracy (%) in sessions 1, 2, and 3.

There was a significant main effect of Familiarity such that participants were more accurate when learning the Familiar language (*M* = 32.95%, *SD* = 16.5) than the Unfamiliar language (*M* = 15.66%, *SD* = 8.36; *F*(1, 97.9) = 21.22, *p* < 0.0001). There was additionally a main effect of Imageability such that words scoring high on imageability (*M* = 30.87%, *SD* = 19.4) were remembered more than those scoring low (*M* = 17.92%, *SD* = 13.4; *F*(1, 194.2) = 62.77, *p* < 0.0001). Lastly, there was a main effect of Session (*F*(1, 9108.7) = 223.43, *p* < 0.0001; see Figure 1), a Familiarity × Imageability interaction (*F*(1, 194.2) = 13.79, *p* < 0.001), and a Familiarity × Session interaction (*F*(2, 9108.7) = 27.92, *p* < 0.0001). Tukey-adjusted pairwise comparisons reveal that the Familiarity × Imageability interaction captures a larger effect of imageability for the Familiar language (*Estimate* = 18.32, *SE* = 2.31, *t*(163.36) = 7.92, *p* < 0.0001) than the Unfamiliar language (*Estimate* = 6.63, *SE* = 2.14, *t*(242.54) = 3.1, *p* = 0.002). The Familiarity × Session interaction reflects the larger improvement from session 1 to 2 for the Familiar language (*Estimate* = 8.63, *SE* = 1.17, *t*(9109.19) = 7.35, *p* < 0.0001) than the Unfamiliar language (*Estimate* = 2.43, *SE* = 1.19, *t*(9102.79) = 2.06, *p* = 0.099), as well as greater memory decay from session 2 to 3 for the Familiar language (*Estimate* = −23.7, *SE* = 1.19, *t*(9117.92) = −19.93, *p* < 0.0001) than the Unfamiliar language (*Estimate* = −11.11, *SE* = 1.19, *t*(9105.36) = −9.35, *p* < 0.0001). In other words, while the more difficult Unfamiliar language was recalled with lower absolute accuracy at each time point (consistent with (de Groot 2006)), it also decayed less over time relative to the Familiar language (consistent with the notion of desirable difficulties, e.g., (Schneider et al. 2002)).^2^

### 3.2. Prior Learning, Language Difficulty, and Vocabulary Retention

Next, we examined the degree to which vocabulary was retained in the final surprise recall test as a function of language difficulty (i.e., imageability and familiarity) as well as levels of prior learning during the Initial and Follow-up Training sessions (session 1 and 2). Accuracy on the final surprise recall test was entered as the outcome variable in a linear mixed-effects model that included fixed effects of Initial Training score, coded as either Not Learned (0 letters correct), Partially Learned (1–3 letters correct), or Learned (4–5 letters correct) in session 1, Follow-up Training score (Not, Partial, or Learned) in session 2, Familiarity (Familiar vs. Unfamiliar), Imageability (High vs. Low), plus all two- and three-way interactions between the two training scores and each of the language difficulty variables. Subject and Item were entered as random effects. Training scores were contrast coded to first compare Learned and Partially Learned words against Not Learned words (‘Learning contrast’) and then Learned words against Partially Learned words (‘Complete contrast’). Table 1 displays parameter estimates and significance values for each effect.

#### 3.2.1. Prior Learning

**Figure 2.**
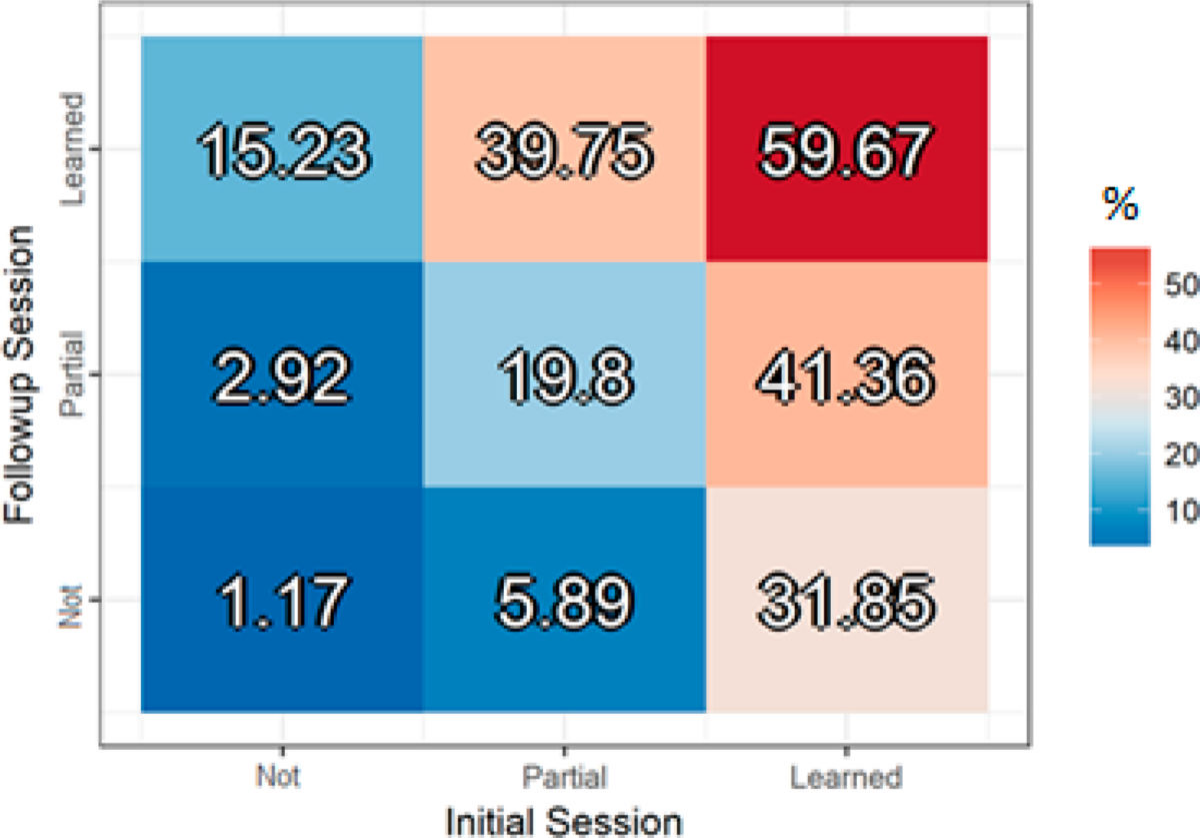
Surprise recall test accuracy (%) based on learning in the initial (first) and follow-up (second) training sessions collapsed across language familiarity and imageability.

There was a main effect of Initial Training score (*F*(2, 2987.7) = 217.83, *p* < 0.0001), such that words that were partially learned during the Initial Training session were later retrieved on the surprise recall test with 15.37% greater accuracy than words that were not learned (*t*(2993.50) = 12.06, *p* < 0.001), and words that were fully learned were later recalled with 22.5% greater accuracy than words that were partially learned (*t*(2979.62) = 11.61, *p* < 0.001). There was a similar, though smaller, main effect of Follow-up Training score (*F*(2, 2988.6) = 134.14, *p* < 0.0001), with partially learned words retrieved on the surprise recall test with 8.39% greater accuracy than not learned words (*t*(2994.19) = 4.49, *p* < 0.001), and fully learned words recalled with 16.86% greater accuracy than partially learned words (*t*(2970.95) = 9.65, *p* < 0.001). There was additionally an interaction between Initial and Follow-up Training scores (*F*(4, 2971.0) = 12.19, *p* < 0.0001), driven primarily by the interaction between the Learning contrast from Initial Training (i.e., Learned + Partially Learned > Not Learned) and the Learning contrast from Follow-up Training (*Estimate* = 11.3, *SE* = 4.6, *p* = 0.014). This interaction captures a super-additive effect, where having at least partial knowledge of the same word in both sessions provides an additional boost to surprise recall beyond the individual effects of each training session. Put differently, words that were partially or fully learned for the first time in the Follow-up session yielded lower final scores compared to those that were at least partially learned and retained across the two training sessions. Figure 2 displays surprise recall accuracy scores based on whether the words were learned, partially learned, or not learned in the Initial and Follow-up Training sessions.

#### 3.2.2. Language Familiarity

**Figure 3.**
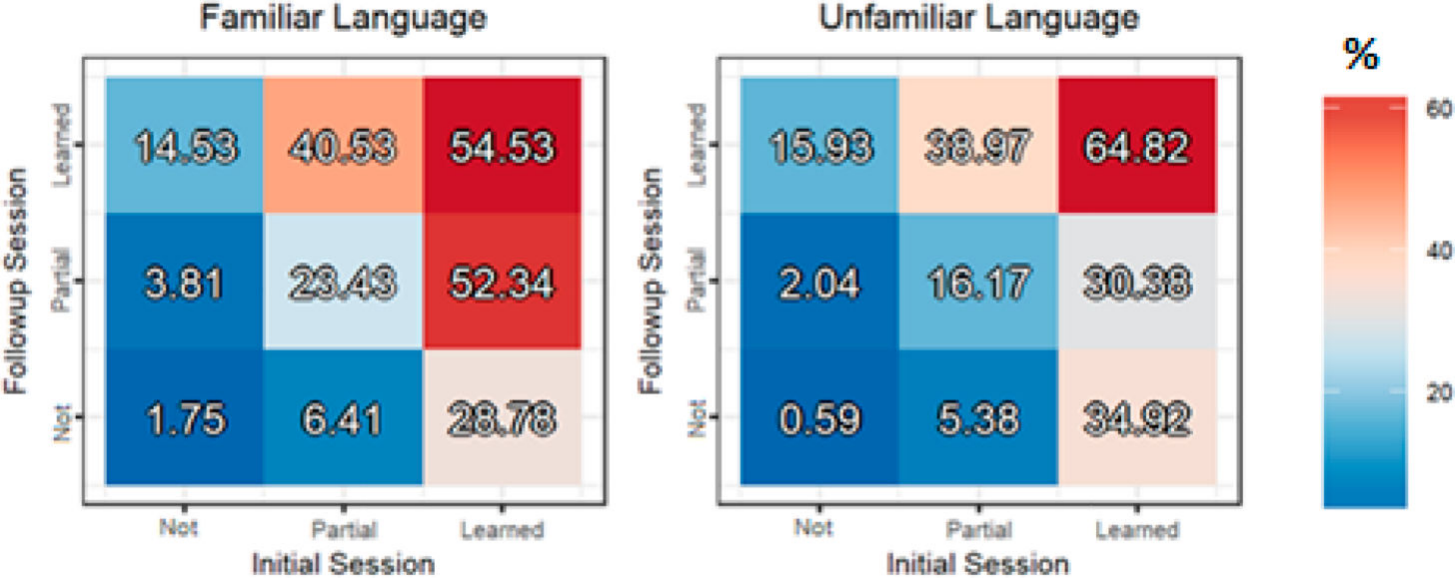
Surprise recall test accuracy (%) based on learning in the initial (first) and follow-up (second) training sessions divided by language group, with the familiar language on the left and the unfamiliar language on the right.

While there was no main effect of Familiarity (*p* = 0.386) on surprise recall accuracy, there was a significant interaction between Familiarity and Follow-up Training score (*F*(2, 2989.1) = 8.68, *p* < 0.001). Unfamiliar words that were fully learned at Follow-up were later recalled with 23.7% greater accuracy than partially learned words, while the additional benefit of full over partial learning for Familiar words was more modest (10%). As can be seen in Figure 3, this was especially the case for words that had been fully learned during Initial Training (right columns), resulting in a significant three-way interaction between Familiarity, Follow-up Training score and Initial Training score (*F*(4, 2970.2) = 3.19, *p* = 0.013). On the other hand, there was a significant benefit of partial recall over no recall for the Familiar language (14.2%, *t*(2989.53) = 5.89, *p* < 0.0001), but not the Unfamiliar language (2.57%; *t*(2995.79) = 0.91, *p* = 0.635). As a result, words that had been fully learned during Initial Training, but then partially forgotten were later recalled with greater accuracy by the Familiar language group compared to the Unfamiliar group (52.34% vs. 30.38%, *t*(2639.62) = 3.09, *p* = 0.002). This was also the case for words that were partially learned during both training sessions (23.43% vs. 16.17%; *t*(602.84) = 2.11, *p* = 0.036). In contrast, those learning the Unfamiliar language were significantly more accurate than the Familiar group for words that were fully learned during both the Initial and Follow-up Training sessions (64.82% vs. 54.53%; *t*(683.28) = 705.06, *p* = 0.004). This pattern is consistent with the idea that greater difficulty may lead to greater retention so long as the word is able to reach a relatively stable state during initial encoding.

#### 3.2.3. Imageability

**Figure 4.**
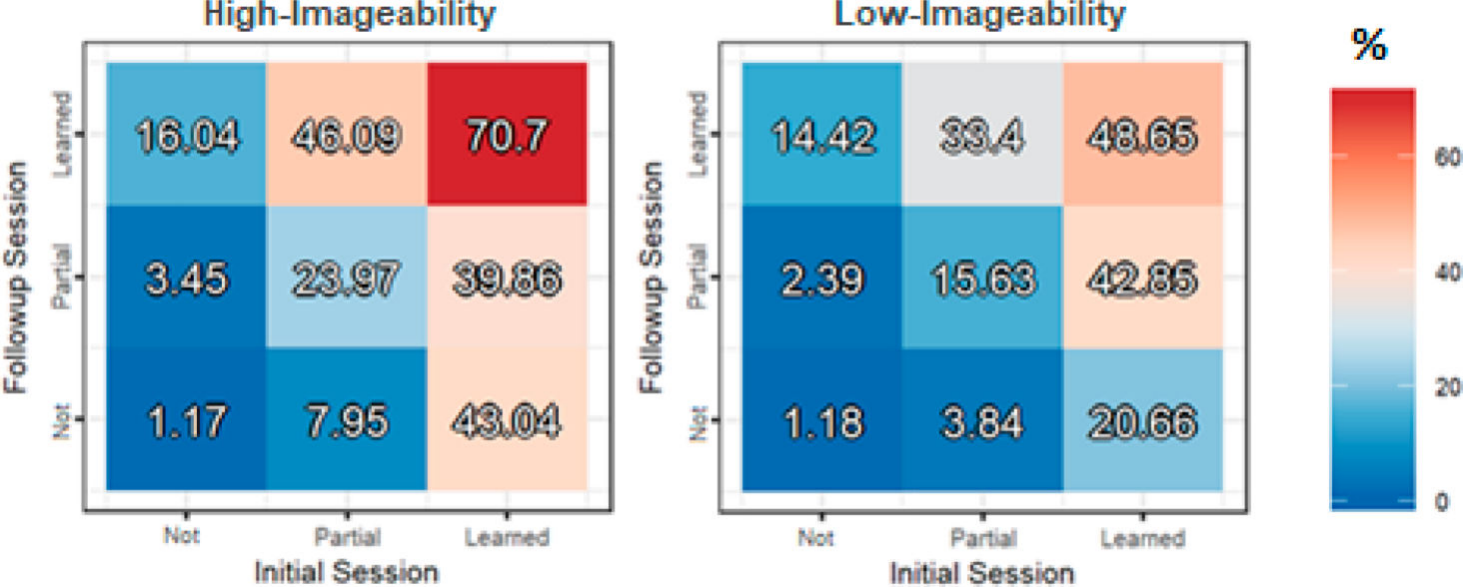
Surprise recall test accuracy (%) based on learning in the initial (first) and follow-up (second) training sessions divided by word imageability, with the high-imageability words on the left and the low-imageability words on the right.

There was a main effect of Imageability (*F*(1, 2954.2) = 34.08, *p* < 0.001), such that accuracy on the surprise recall test was 7.69% higher for high-imageability words than low-imageability words overall. There was additionally a significant Imageability × Initial Training score interaction (*F*(2, 2958.4) = 8.91, *p* < 0.001), such that high-imageability words that were fully learned during Initial Training were eventually recalled with 25.2% greater accuracy than partially learned words, while the additional benefit of fully learning low-imageability words was more modest (19.7%). A similar interaction was found between Imageability and Follow-up Training score, with a greater difference between full and partial learning for high (21.8%) than low-imageability words (11.8%) (*F*(2, 2957.5) = 4.94, *p* = 0.007). As can be seen in Figure 4, the interaction between Imageability and Follow-up Training score was especially pronounced for words that had been fully learned during Initial Training (right columns), resulting in a significant three-way interaction between Imageability, Follow-up Training score, and Initial Training score (*F*(4, 2956.3) = 2.95, *p* = 0.019). Note that this pattern is the opposite of what was found for the Familiarity manipulation, where the added benefit of fully learning a word during Follow-up (vs. partial) was greater for the more difficult (unfamiliar) words, whereas here we observe a greater benefit for easier (high-imageability) words. Put differently, when a high-imageability word was fully learned in Initial Training, even partially forgetting the word during Follow-up led to substantially lower final recall compared to consistently remembered words. Final recall of initially learned low-imageability words, on the other hand, benefited similarly from partially and fully remembered words during Follow-up Training compared to completely forgotten words.

## 4. Discussion

We began by asking the question of whether language difficulty helps or hinders the retention of foreign language vocabulary. In light of mixed results in past studies, we proposed that the effect of language difficulty on vocabulary retention may be partially moderated by how stably words are encoded in memory during prior learning. To investigate this possibility, we manipulated two variables known to influence the difficulty of initial vocabulary acquisition (word imageability and similarity to the native language) and observed their influence on eventual retention after accounting for the degree of learning during two previous training sessions.

In order to directly compare the present results with those of similar past studies, we examined the effects of imageability and native language similarity (i.e., ‘familiarity’) on recall at each time point without accounting for the effects of prior learning on subsequent retention. Consistent with de Groot and Keijzer (2000) and de Groot (2006), we observed that recall was greater for ‘easier’ words (high-imageability and familiar) than more difficult words (low-imageability and unfamiliar) at each of the three time points. We additionally observed that improvement from the initial training session to the follow-up session (week 1 to week 2) was greater for the easier words than for the more difficult words. However, when participants returned two weeks later for a surprise recall test, the amount of memory loss was significantly greater for the easier, familiar words compared to the more difficult, unfamiliar words. While high-imageability and low-imageability words were forgotten to similar extents, the relatively greater retention of unfamiliar words over familiar words suggest that certain types of language difficulty may indeed buffer against memory decay, even without accounting for prior learning. This pattern is consistent with the findings of Schneider et al. (2002), who observed that introducing task difficulty through more challenging testing procedures resulted in greater retention of novel vocabulary over time.

Turning to the effects of prior learning on final recall, we observed that performance on both the initial and follow-up training sessions predicted later retention. Interestingly, we observed that the level of learning during the initial session was more predictive of final recall than performance during the follow-up session, despite the fact that the latter occurred closer in time to the final test. For instance, words that were initially fully learned, but then completely forgotten during the follow-up training were ultimately recalled with 31.9% accuracy, as compared to 15.2% for words that were not successfully learned during initial training, but then fully learned during the follow-up session. This suggests that the depth of initial encoding plays a significant role in eventual retention. We additionally observed an additive effect of the two training sessions—recalling a word in both sessions boosted ultimate recall beyond the independent effects of learning in the initial and follow-up training sessions alone.

Most pertinent to the current investigation, we observed that the interactive effects of initial and follow-up training on final recall were moderated by language difficulty. First, we observed that words that were only partially recalled during either training session were later recalled with significantly greater accuracy for the easier, familiar language relative to the unfamiliar language. One possibility is that relatively unstable memories are more easily retrieved when participants can rely on existing knowledge regarding phonotactic rules to recreate probable word-forms (Gathercole et al. 1999). Indeed, the fact that words that were fully recalled during both training sessions resulted in relatively similar levels of final recall accuracy as those that were only partially recalled during follow-up may indicate that even ‘fully learned’ familiar words were in fact still in a relatively unstable memory state. Being able to rely on prior knowledge of phonological regularities may have thus contributed to the overall advantage of familiar words over unfamiliar words, particularly when they were recalled with only partial accuracy during training.

Words that were fully learned in both initial and follow-up training sessions, on the other hand, were more accurately recalled during the final test for the more difficult unfamiliar language. In other words, we observed an effect of ‘desirable difficulties,’ but only when the words had reached a relatively stable memory state during early training (i.e., the ‘P’ state as conceptualized by Atkinson (1972)). This finding may help resolve some seemingly inconsistent past results regarding the effects of task difficulty on vocabulary retention. As noted previously, Schneider et al. (2002) observed that introducing task difficulty improved eventual retention, while de Groot and Keijzer (2000) and de Groot (2006) found that more difficult words were most easily forgotten. Schneider and colleagues proposed that these variable effects may be explained by the difference between introducing difficulty in the testing procedure or process, as in their study (e.g., forward vs. backward translation), versus difficulty of the words themselves, as in de Groot and Keijzer’s studies (e.g., imageability, typicality). However, the reason for this distinction is not yet clear. The present findings may help offer a possible explanation. As proposed in the *desirable difficulty* literature (e.g., Bjork and Bjork 2011), challenging learning and testing conditions may force participants to engage in more intensive and flexible processing of the materials, leading to more durable memories. However, in the absence of external pressure to process material with greater depth, words that are relatively more difficult may be less likely to reach permanent states of encoding, as proposed by de Groot (2006). However, in cases where words *do* manage to reach relatively stable states of encoding during training, task difficulty may indeed enhance retention, even when it pertains to the word itself rather than the testing procedure.

The effects of language familiarity suggest that language difficulty influences vocabulary retention differently depending on initial levels of encoding. However, some open questions remain. Namely, why did we not observe a similar *desirable difficulty* effect for low-imageability words that were correctly recalled during both training sessions? One possibility is that the difference between the effects of language familiarity and word imageability stems from the fact that the former was manipulated between subjects, while the latter was manipulated within-subjects. It may be the case that individuals engage in a deeper level of processing when the entire corpus of material to be learned is relatively difficult, but not when some words are more easily acquired than others. In the case of the latter, participants may prioritize memorization of the relatively easier words at the expense of more difficult words. An alternative, or additional, explanation is that the presumably ‘fully learned’ low-imageability words (that is, those that were accurately recalled during both training sessions) were in fact still in an unstable memory state. As in the case of ‘fully learned’ familiar words, we once again observed that the advantage of complete accuracy during both training sessions on final recall of low-imageability words was not substantially greater than that of words that were partially forgotten during follow-up (with final accuracy scores of 48.7% vs. 42.9%, respectively). Compare this to the previously discussed advantage of fully learned unfamiliar words over partially learned unfamiliar words (64.8% vs. 30.4%, respectively), as well as fully learned high-imageability words over partially learned high-imageability words (70.7% vs. 39.9%, respectively). The variable effects observed for imageability and familiarity may additionally suggest that linguistically driven challenges can have distinct consequences for long-term retention depending on whether difficulty is introduced for encoding meaning (i.e., semantic retrieval of visual imagery) versus form (i.e., phonotactic similarity to known languages). For instance, it may be the case that the advantages initially conferred by greater semantic access are relatively durable over time, whereas the initial difficulty of learning unfamiliar forms may (at times) encourage learners to engage in more elaborate processing, resulting in greater retention. According to the Revised Hierarchical Model of bilingual representation (Kroll and Stewart 1994), L2 words initially access conceptual representations via their L1 translations, and language mastery is attained through the strengthening of direct L2-to-concept connections. The advantages of imageable/concrete words for both acquisition and retention may therefore lie in the vividness of conceptual representations that are coactivated during L2 exposure, facilitating the process of linking novel words to their referents. In contrast, similarity to familiar word-forms is likely to strengthen L2-to-L1 connections, which can promote initial acquisition, but potentially inhibit the transition to direct conceptual mediation. Because learners cannot rely as much on existing phonotactic knowledge when encoding and retrieving unfamiliar word-forms, they may, in some cases, engage in more semantically mediated strategies, resulting in more stable, conceptually grounded representations of novel words.

The effects of level of representation (e.g., meaning vs. form), procedural differences (e.g., manipulating difficulty between- vs. within-subject), as well as differences in the stability of presumably ‘fullylearned’stimulicouldbeaddressedinfutureresearchtogainamorecomprehensiveunderstanding of how language difficulty influences word learning. Follow-up studies will also be needed to determine the role of individual differences, both in terms of experiences that would be directly relevant to the study materials, as well as more general capacities that would likely influence the degree to which initial challenges will be helpful or harmful for long-term learning. One limitation of the current experiment is that our manipulation of imageability was based on norms generated by individuals other than the participants themselves—given that imageability is likely to be dependent on one’s personal experience with a particular referent (Stadthagen-Gonzalez and Davis 2006), follow-up studies would benefit from asking participants to provide imageability ratings of the stimuli, thereby confirming the effectiveness of the manipulation. Likewise, though our manipulation of familiarity was based on objective similarity to phonotactic regularities found in English, individual knowledge and experience associated with English and other known languages will likely contribute to variable findings across participants. Future research may additionally examine whether individual differences in cognitive abilities such as working memory moderate the impact of language difficulty. In many cases, individuals with lower capacity may be especially likely to experience initial difficulties, which may either promote or hinder long-term gains depending on the degree to which executive function is taxed (Bui et al. 2013; Agarwal et al. 2017; Kachergis et al. 2017).

In conclusion, we provide evidence that the effect of language difficulty on the long-term retention of foreign vocabulary can vary as a function of how well novel word-forms are learned during initial training. While more difficult words may be less likely to reach stable levels of encoding (as suggested by de Groot 2006), those that do are even more likely to be retained over time than more easily acquired words—a pattern that is consistent with the *desirable difficulty* effects obtained by Schneider et al. (2002). Our findings thus indicate that establishing a solid foundation of knowledge during early stages of vocabulary acquisition is critical to allow learners to not only overcome initial difficulties, but thrive when faced with later challenges. While more work is needed to establish potential boundaries, our findings suggest that when it comes to remembering a foreign language, what does not kill you can make you stronger.

## Figures and Tables

**Table 1.** Parameter estimates for linear mixed effect regression model of Initial Training score, Follow-up Training score, and Language on surprise recall performance.

	Estimate	SE	Df	t	p	
Intercept	9.90	1.50	105.60	6.56	<0.001	***
Initial:Learn	17.80	2.40	2992.97	7.52	<0.001	***
Initial:Complete	20.50	4.20	2982.22	4.93	<0.001	***
Followup:Learn	12.00	2.00	2995.05	6.04	<0.001	***
Followup:Complete	16.50	3.10	2974.32	5.27	<0.001	***
Familiarity	0.70	2.00	78.37	0.36	0.717	
Imageability	4.50	1.10	2951.47	4.25	<0.001	***
Initial:Learn|Followup:Learn	11.30	4.60	2981.10	2.46	0.014	*
Initial:Complete|Followup:Learn	0.10	7.90	2974.25	0.02	0.988	
Initial:Learn|Followup:Complete	7.70	6.30	2957.39	1.21	0.227	
Initial:Complete|Followup:Complete	1.30	9.30	2952.61	0.14	0.888	
Initial:Learn|Familiarity	−0.50	2.70	2995.89	−0.17	0.863	
Initial:Complete|Familiarity	−4.20	4.60	2981.48	−0.92	0.359	
Followup:Learn|Familiarity	1.90	2.30	2988.85	0.83	0.404	
Followup:Complete|Familiarity	−7.90	3.40	2977.97	−2.33	0.02	*
Initial:Learn|Imageability	11.70	2.50	2952.53	4.62	<0.001	***
Initial:Complete|Imageability	10.70	4.40	2955.08	2.43	0.015	*
Followup:Learn|Imageability	0.60	2.10	2954.66	0.26	0.792	
Followup:Complete|Imageability	4.90	3.20	2964.57	1.52	0.129	
Initial:Learn|Followup:Learn|Familiarity	8.70	5.10	2990.06	1.71	0.088	
Initial:Complete|Followup:Learn|Familiarity	8.60	8.40	2975.03	1.02	0.308	
Initial:Learn|Followup:Complete|Familiarity	−15.80	6.60	2958.09	−2.41	0.016	*
Initial:Complete|Followup:Complete|Familiarity	−26.60	8.80	2947.61	−3.02	0.003	**
Initial:Learn|Followup:Learn|Imageability	−4.60	4.90	2954.56	−0.94	0.347	
Initial:Complete|Followup:Learn|Imageability	−19.30	8.10	2952.95	−2.37	0.018	*
Initial:Learn|Followup:Complete|Imageability	14.10	6.20	2958.59	2.26	0.024	*
Initial:Complete|Followup:Complete|Imageability	20.70	8.30	2958.09	2.50	0.013	*

*** *p* < 0.001

** *p* < 0.01

* *p* < 0.05

Initial:Learn = Learned + Partially Learned > Not Learned contrast in the Initial Training Session (session 1); Initial:Complete = Learned > Partially Learned contrast in the Initial Training Session (session 1); Followup:Learn = Learned + Partially Learned > Not Learned contrast in the Follow-up Training Session (session 2); Followup:Complete = Learned > Partially Learned contrast in the Follow-up Training Session (session 2).

## Footnotes

1 Note that de Groot and Keijzer (2000) characterized the measure as concreteness, but the stimuli were in fact categorized based on imageability ratings.

2 Including either a categorical (Monolingual vs. Not) or continuous (Non-English Proficiency) variable as a predictor did not alter the significance of the reported effects, and there were no significant effects or interactions with Language Status (*p* > 0.05). Though including Non-English Proficiency as a continuous predictor did yield a significant three-way interaction between Proficiency, Imageability, and Session (F(2, 9103) = 3.58, *p* = 0.028), Holm–Bonferroni-adjusted follow-up tests revealed no significant interactions between Proficiency and Imageability in Session 1 (*p* > 0.9), Session 2 (*p* = 0.24), or Session 3 (*p* = 0.14). Similarly, while there was a significant three-way interaction between Proficiency, Familiarity, and Session (*F*(2, 9111.1) = 4.28, *p* = 0.014), the lower-order effects of Proficiency did not approach significance in any of the sessions (*p* > 0.9).
